# Impaired Fasting Glucose and Diabetes Are Related to Higher Risks of Complications and Mortality Among Patients With Coronavirus Disease 2019

**DOI:** 10.3389/fendo.2020.00525

**Published:** 2020-07-10

**Authors:** Jiaoyue Zhang, Wen Kong, Pengfei Xia, Ying Xu, Li Li, Qin Li, Li Yang, Qi Wei, Hanyu Wang, Huiqing Li, Juan Zheng, Hui Sun, Wenfang Xia, Geng Liu, Xueyu Zhong, Kangli Qiu, Yan Li, Han Wang, Yuxiu Wang, Xiaoli Song, Hua Liu, Si Xiong, Yumei Liu, Zhenhai Cui, Yu Hu, Lulu Chen, An Pan, Tianshu Zeng

**Affiliations:** ^1^Department of Endocrinology, Union Hospital, Tongji Medical College, Huazhong University of Science and Technology, Wuhan, China; ^2^Hubei Provincial Clinical Research Center for Diabetes and Metabolic Disorders, Wuhan, China; ^3^Department of Epidemiology and Biostatistics, School of Public Health, Tongji Medical College, Huazhong University of Science and Technology, Wuhan, China; ^4^Department of Endocrinology, The Fifth Hospital of Wuhan, Wuhan, China; ^5^Department of Endocrinology, Wuhan Wuchang Hospital, Wuchang Hospital Affiliated to Wuhan University of Science and Technology, Wuhan, China; ^6^Department of Endocrinology, General Hospital of the Yangtze River Shipping, Wuhan, China; ^7^Department of Endocrinology, Hankou Hospital of Wuhan City, Wuhan, China; ^8^Department of Endocrinology, Red Cross Hospital of Wuhan City, Wuhan, China; ^9^Institute of Hematology, Union Hospital, Tongji Medical College, Huazhong University of Science and Technology, Wuhan, China; ^10^Hubei Clinical and Research Centre of Thrombosis and Haemostasis, Wuhan, China

**Keywords:** cohort study, coronavirus, COVID-19, diabetes, hyperglycemia, impaired fasting glucose, severe acute respiratory coronavirus 2 (SARS-CoV-2)

## Abstract

**Background:** Diabetes correlates with poor prognosis in patients with COVID-19, but very few studies have evaluated whether impaired fasting glucose (IFG) is also a risk factor for the poor outcomes of patients with COVID-19. Here we aimed to examine the associations between IFG and diabetes at admission with risks of complications and mortality among patients with COVID-19.

**Methods:** In this multicenter retrospective cohort study, we enrolled 312 hospitalized patients with COVID-19 from 5 hospitals in Wuhan from Jan 1 to Mar 17, 2020. Clinical information, laboratory findings, complications, treatment regimens, and mortality status were collected. The associations between hyperglycemia and diabetes status at admission with primary composite end-point events (including mechanical ventilation, admission to intensive care unit, or death) were analyzed by Cox proportional hazards regression models.

**Results:** The median age of the patients was 57 years (interquartile range 38–66), and 172 (55%) were women. At the time of hospital admission, 84 (27%) had diabetes (and 36 were new-diagnosed), 62 (20%) had IFG, and 166 (53%) had normal fasting glucose (NFG) levels. Compared to patients with NFG, patients with IFG and diabetes developed more primary composite end-point events (9 [5%], 11 [18%], 26 [31%]), including receiving mechanical ventilation (5 [3%], 6 [10%], 21 [25%]), and death (4 [2%], 9 [15%], 20 [24%]). Multivariable Cox regression analyses showed diabetes was associated increased risks of primary composite end-point events (hazard ratio 3.53; 95% confidence interval 1.48–8.40) and mortality (6.25; 1.91–20.45), and IFG was associated with an increased risk of mortality (4.11; 1.15–14.74), after adjusting for age, sex, hospitals and comorbidities.

**Conclusion:** IFG and diabetes at admission were associated with higher risks of adverse outcomes among patients with COVID-19.

## Introduction

The coronavirus disease 2019 (COVID-19) pandemic, a public health emergency of international concern, had affected more than 8.1 million cases and caused over 440,000 deaths globally by June 18, 2020 (1). People of all ages can be infected, whereas older people and those with underlying diseases were more likely to develop severe illness (2, 3).

The prevalence of diabetes among patients with COVID-19 varied in different studies. Studies in Chinese patients reported prevalence rates ranged from 5.3 to 8.2% (3–5), while a recent study in 5,700 patients with New York reported that 33.8% had diabetes (6). Previous studies have reported that diabetes and uncontrolled glycemia were significant predictors of severity and mortality in patients infected with lower respiratory tract infections (7, 8), 2009 pandemic influenza A (H1N1) (9), severe acute respiratory syndrome coronavirus (SARS-CoV) (10), and Middle East respiratory syndrome coronavirus (MERS-CoV) (11, 12). However, it remains controversial whether diabetes is related to adverse outcomes among patients with COVID-19 (13, 14). Most studies reported that diabetes was associated with higher risks for severe events and mortality (3–5, 15–17), whereas others showed no clear association (18, 19). The inconsistency may be related to the varied sample size, different populations, and different degrees of confounding adjustment. Hyperglycemia has been widely accepted to be harmful to the control of infection. A recent study of 7,337 cases with COVID-19 in China found that well-controlled blood glucose (3.9–10.0 mmol/L) was associated with markedly lower mortality compared to individuals with poorly controlled BG (>10.0 mmol/L) (20). On the other hand, an overly rigid glucose control might also increase the risk of severe hypoglycemia, which can also lead to an increased mortality (21). Nevertheless, no study has specifically examined the prevalence of impaired fasting glucose (IFG) in patients with COVID-19 and whether prediabetes condition was a risk factor.

Many studies have reported the clinical features of patients with COVID-19 in different countries (2, 5, 6, 16, 22–24), while few has specifically compared the clinical characteristics of COVID-19 in patients with and without diabetes. And also, the clinical characteristics of IFG patients with COVID-19 are obscure until now. It is unclear whether the differences in those characteristics including laboratory markers may explain the increased risks of adverse outcomes related to prediabetes and diabetes. Therefore, we analyzed clinical and laboratory characteristics, as well as treatment and prognosis of hospitalized patients with COVID-19 by diabetes and hyperglycemia status at admission in Wuhan city. We hypothesized that diabetes and IFG were related to increased risks of adverse outcomes and differences in clinical features could mediate the associations.

## Materials and Methods

### Study Design and Participants

In this multicenter, retrospective cohort study, we recruited hospitalized patients with COVID-19 from six departments of five hospitals in Wuhan from Jan 1 to Mar 17, 2020. All hospitals were designated to treat patients with COVID-19, including the Department of Infectious Disease and the Department of Oncology of the Union Hospital of Tongji Medical College, Huazhong University of Science and Technology, the Departments of Endocrinology in the following four hospitals: Fifth Hospital of Wuhan, Wuhan Wuchang Hospital Affiliated to Wuhan University of Science and Technology, General Hospital of the Yangtze River Shipping, and Wuhan Hankou Hospital. Those six departments were temporarily converted into isolated wards for patients with COVID-19. We only had access to the data of the six departments that the investigators were in charge of, and thus data from other departments in the five hospitals were not available.

COVID-19 was diagnosed according to the Diagnosis and Treatment Scheme for the Novel Coronavirus Pneumonia released by the National Health Commission of China [Supplementary Table 1; (3)], and the severity status of the patients were classified as non-severe and severe types [Supplementary Table 2; (25)]. We only included cases with positive results for severe acute respiratory coronavirus 2 (SARS-CoV-2) virus by real-time reverse transcriptase-polymerase chain reaction (RT-PCR) assay of nasal and pharyngeal swab specimens, or positive serum specific IgM and IgG antibody.

The study was approved by the Ethics Committee of the Union Hospital, Tongji Medical College, Huazhong University of Science and Technology. Informed consent was waived by using anonymous clinical data in this retrospective study.

### Data Collection

We extracted data from electronic medical records for demographics, clinical, laboratory, and radiological characteristics, treatment, and outcomes for all patients with COVID-19. Two researchers independently reviewed and double checked the data collection forms.

General characteristics (age, sex, and comorbidities) and clinical symptoms and signs at admission were recorded. Vital signs (respiratory rate, blood pressure) were measured, height and weight were self-reported. Comprehensive laboratory test results were compiled within 3 days of admission and before steroid therapy. If fasting glucose concentrations were measured multiple times after admission, we only used the first one to represent the glycemic status at the time of admission. The treatment regimens for COVID-19 and other comorbidities were also extracted from the medical records.

Diabetes was diagnosed as fasting plasma glucose ≥7.0 mmol/L, or self-reported physician-diagnosed diabetes or anti-diabetic medication use; IFG was defined as glucose levels between 5.6 and 6.9 mmol/L. Patients with fasting glucose levels below 5.6 mmol/L were considered as having normal fasting glucose (NFG). Patients without previous diagnosis of diabetes while presenting with plasma fasting glucose ≥7.0 mmol/L at hospital admission was considered as new-diagnosed diabetes.

The primary composite endpoints included mechanical ventilation, admission to intensive care unit (ICU), or death. Other endpoints were also recorded, including acute respiratory distress syndrome (ARDS), septic shock, acute kidney injury, cardiac injury, rhabdomyolysis, diabetic ketoacidosis, hyperosmolar hyperglycemic state, and hypoglycemic coma. Details of the definitions of the outcomes are provided in the Supplementary Material.

Laboratory test results were compiled including standard blood counts, blood biochemistry [including renal and liver function, creatine kinase, fasting plasma glucose, lactate dehydrogenase (LDH), and lipid profiles], urine routine test, coagulation profile, procalcitonin, C-reactive protein (CRP), erythrocyte sedimentation rate (ESR), myocardial enzyme spectrum and routine bacterial, fungal, and viral examinations. Additional data were collected including medical imaging, treatment regimens [e.g., antiviral and antibacterial drugs, systemic corticosteroid, immunoglobulin G, respiratory support (e.g., nasal tube, high-flow nasal cannula, non-invasive, and invasive mechanical ventilation)], and prognosis (discharged or death). Anti-diabetic agents during hospitalization were recorded.

### Statistical Analysis

Descriptive statistics included counts and proportions for categorical variables and median (IQR) for continuous variables. Comparisons across the three categories (diabetes, IFG, NFG) were performed using Kruskal Wallis test for continuous variables and chi-square or Fisher's exact test for categorical variables as appropriate. Logistical regression was performed to evaluate the association between diabetes status and severity of COVID-19 with adjustment for age, sex, hospitals and other comorbidities. Time to a composite endpoint was investigated using survival analysis by a Kaplan- Meier plot and compared by the log-rank test. Furthermore, Cox proportional hazards regression models were used to evaluate the association between glycemic status and outcomes with adjustment for age, sex, hospitals and other comorbidities. To further investigate whether the associations were mediated by certain laboratory markers, we classified the markers into different categories (such as blood biochemistry, inflammatory markers, metabolic markers) and chose a marker with the strongest association with the outcomes as a representative of the category and included in the final model. This was used to avoid over-adjustment for many markers with collinearity in the model with limited sample size. There were missing values for some laboratory tests and were treated as missing indicators in the regression models to retain maximum sample size. Sensitivity analysis was performed by removing patients with new-diagnosed diabetes to exclude the possibility of stress induced hyperglycemia. Statistical analyses were performed with SAS 9.4, and statistical significance was set at 2-tailed *p* < 0.05.

## Results

### General Information

By Mar 17, 2020, 729 patients with pneumonia were admitted to the six departments of five hospitals. Among them, the following patients were excluded from the analyses: 316 suspected cases without positive RT-RCP tests, 80 patients who were still in hospital until Mar 17, 2020, 21 patients without intact information of clinical outcomes because of transferring to other hospitals. Therefore, 312 patients were included in the final analysis. Among them, 84 (27%) had diabetes, 62 (20%) had IFG, and 166 (53%) had NFG. Among the 84 patients with diabetes, 57 had fasting glucose levels ≥7.0 mmol/L, including 30 without and 27 with a known history of diabetes.

The median age of the 312 patients was 57 years (interquartile range 38–66), and 172 (55%) were female (Table 1). Comparing to patients with NFG, patients with IFG and diabetes were older and more likely to be men. As expected, patients with IFG and diabetes were more likely to have other comorbidities, including hypertension, coronary heart diseases, chronic kidney disease, and cerebrovascular disease.

**Table 1:** Demographics and clinical symptoms of patients with COVID-19 according to diabetes status.

	**All patients (*n* = 312)**	**Diabetes (*n* = 84)**	**IFG (*n* = 62)**	**NFG (*n* = 166)**	***P*-value**
**Age**	57 (38-66)	62 (55-70)	62 (43-66)	46 (34-64)	<0.001
**Sex, females**	172 (55%)	34 (40%)	28 (45%)	110 (66%)	<0.001
**Any comorbidities**	115 (37%)	45 (54%)	28 (45%)	42 (25%)	<0.001
Hypertension	89 (29%)	42 (50%)	22 (35%)	25 (15%)	<0.001
Coronary heart disease	22 (7%)	13 (15%)	4 (6%)	5 (3%)	0.002
Chronic lung disease	12 (4%)	3 (4%)	2 (3%)	7 (4%)	>0.99
Chronic liver disease	6 (2%)	0 (0%)	2 (3%)	4 (2%)	0.24
Chronic kidney disease	8 (3%)	4 (5%)	3 (5%)	1 (1%)	0.04
Cerebrovascular disease	15 (5%)	8 (10%)	4 (6%)	3 (2%)	0.01
Cancer	12 (4%)	2 (2%)	4 (6%)	6 (4%)	0.45
**Signs and symptoms at admission**					
Fever	268 (86%)	75 (89%)	57 (92%)	136 (82%)	0.09
Fatigue	181 (58%)	53 (63%)	31 (50%)	97 (58%)	0.28
Cough	264 (85%)	75 (89%)	51 (82%)	138 (83%)	0.38
Myalgia	70 (22%)	17 (20%)	13 (21%)	40 (24%)	0.75
Redness of the eyes	0 (0%)	0 (0%)	0 (0%)	0 (0%)	NA
Dyspnea	145 (46%)	56 (67%)	34 (55%)	55 (33%)	<0.001
Headache	37 (12%)	8 (10%)	6 (10%)	23 (14%)	0.51
Rhinorrhea	5 (2%)	1 (1%)	0 (0%)	4 (2%)	0.60
Chest pain	139 (45%)	46 (55%)	26 (42%)	67 (40%)	0.09
Diarrhea	78 (25%)	27 (32%)	17 (27%)	34 (20%)	0.12
Nausea and vomiting	41 (13%)	13 (15%)	9 (15%)	19 (11%)	0.63
Palpitation	34 (11%)	8 (10%)	8 (13%)	18 (11%)	0.81
Loss of appetite	179 (57%)	64 (76%)	30 (48%)	85 (51%)	<0.001
polypnea	30 (10%)	13 (15%)	8 (13%)	9 (5%)	0.02
Hypoxemia	120 (38%)	50 (60%)	30 (48%)	40 (24%)	<0.001

*Data are shown as median (IQR) and n (%)*.

*P-values were derived from χ^2^ test, Fisher's exact test, Kruskal Wallis test when appropriate*.

*COVID-19, 2019 novel coronavirus; IFG, impaired fasting glucose; NFG, normal fasting glucose*.

### Clinical Symptoms and Signs

The common symptoms at hospital admission included fever (268 [86%]), cough (264 [85%]), fatigue (181 [58%]), loss of appetite (179 [57%]), dyspnea (145 [46%]), and chest pain 139 (45%; Table 1). Among the symptoms, patients with diabetes were more likely to have dyspnea (67 vs. 33%), appetite loss (76 vs. 51%), and polypnea (15 vs. 5%) compared to those with NFG. The median time from onset of symptoms to hospital admission was 8 days (5–11 days), and no significant differences were found across the three groups (Table 1).

### Laboratory Tests and Imaging Examinations

At admission, patients with diabetes and IFG had higher neutrophils, while lower lymphocytes, eosinophils, and platelets compared with patients with NFG; thus the proportions of lymphocytopenia, eosinopenia, and thrombocytopenia were higher among patients with diabetes and IFG (Table 2). No significant differences were found in the levels of leucocyte, monocytes, basophils, and hemoglobin (Table 2).

**Table 2:** Laboratory results and radiologic findings of patients with COVID-19 according to diabetes status.

	**All patients (*n* = 312)**	**Diabetes (*n* = 84)**	**IFG (*n* = 62)**	**NFG (*n* = 166)**	***P*-value**
**Blood routine**					
Leucocytes (×10^9^ per L)	4.8 (3.5–6.5)	5.0 (3.6–6.9)	4.6 (3.4–6.6)	4.6 (3.5–6.1)	0.34
Neutrophils (×10^9^ per L)	3.0 (2.1–4.6)	3.6 (2.4–5.7)	3.1 (2.1–4.8)	2.7 (2.0–3.7)	0.005
Increased (>6.3)	30 (11%)	14 (18%)	7 (11%)	9 (6%)	0.047
Decreased (<1.8)	43 (15%)	7 (9%)	11 (18%)	25 (17%)	
Lymphocytes (×10^9^ per L)	1.1 (0.8–1.4)	0.8 (0.6–1.2)	1.0 (0.7–1.5)	1.2 (0.9–1.6)	<0.001
Increased (>3.2)	2 (1%)	0 (0%)	0 (0%)	2 (1%)	<0.001
Decreased (<1.1)	146 (51%)	56 (72%)	35 (57%)	55 (38%)	
Eosinophils (×10^9^ per L)	0.01 (0–0.06)	0 (0–0.03)	0.01 (0–0.03)	0.03 (0–0.08)	<0.001
Decreased (<0.02)	152 (53%)	54 (68%)	38 (62%)	60 (41%)	<0.001
Basophils (×10^9^ per L)	0.01 (0–0.02)	0.01 (0–0.03)	0.01 (0–0.01)	0.01 (0.01–0.02)	0.013
Hemoglobin (g/L)	126 (116–136)	128 (116–139)	128 (114–137)	124 (116–135)	0.44
Platelets (×10^9^ per L)	175 (129–234)	161 (113–200)	161 (129–203)	199 (144–247)	<0.001
Increased (>350)	13 (5%)	2 (3%)	2 (4%)	9 (6%)	0.02
Decreased (<120)	51 (19%)	22 (29%)	13 (23%)	16 (12%)	
**Blood biochemistry**					
Alanine aminotransferase (U/L)	23 (16-35)	28 (19-39)	26 (18-43)	21 (15-30)	0.002
Increased (>35.0)	70 (25%)	24 (30%)	21 (34%)	25 (17%)	0.01
Aspartate aminotransferase (U/L)	27 (20-40)	34 (20-47)	33 (23-46)	23 (19-32)	<0.001
Increased (>40.0)	69 (24%)	26 (33%)	21 (35%)	22 (15%)	0.002
Glutamate transpeptidase (U/L)	23 (16-39)	25 (17-40)	36 (17.0–55.0)	20 (14-32)	<0.001
Albumin (g/L)	36.5 (33.1–40.8)	35.7 (33.0–38.7)	35.6 (33.1–40.5)	37.8 (34.2–41.1)	0.03
Decreased (<35.0)	103 (36%)	33 (42%)	28 (46%)	42 (29%)	0.04
eGFR (mL/min/1.73m^2^)	95.3 (82.2–108.6)	91.7 (70.9–99.8)	91.4 (78.7–101.5)	99.6 (90.5–114.5)	<0.001
Decreased (<90)	100 (36%)	38 (49%)	28 (47%)	34 (24%)	<0.001
Cystatin C (mg/L)	0.9 (0.8–1.1)	1.1 (0.9–1.4)	0.9 (0.8–1.3)	0.8 (0.7–1.1)	<0.001
Increased (>1.15)	52 (24%)	19 (33%)	14 (30%)	19 (17%)	0.03
Creatine kinase (U/L)	69.9 (45.5–127)	90 (49–160)	93 (50–179)	59 (43–100)	0.002
Increased (>140.0)	52 (22%)	19 (31%)	15 (28%)	18 (15%)	0.007
Decreased (<26.0)	4 (2%)	0 (0%)	3 (6%)	1 (1%)	
Troponin I (ng/L)	5.35 (2.6–10.0)	10 (2.6–10.0)	10 (3.3–19.4)	4.55 (1.2–10.0)	0.03
Increased (>26.2)	11 (10%)	4 (14%)	4 (19%)	3 (5%)	0.15
CKMB (ng/ml)	2.0 (0.6–2.9)	2.0 (1.4–2.6)	1.5 (0.8–2.9)	2.0 (0.5–3.2)	0.88
Increased (≥6.6)	6 (4%)	2 (5%)	0 (0%)	4 (4%)	0.64
Brain natriuretic peptide (pg/mL)	42.8 (10.0–92.2)	82.0 (14.1–91.6)	202.8 (68.4–327.3)	11.9 (10.0–49.4)	0.10
Increased (≥27.5)	8 (50%)	3 (60%)	3 (100%)	2 (25%)	0.16
Total carbon dioxide (mmol/L)	23.6 (21.2–25.5)	23.4 (19.7–25.3)	22.0 (19.9–24.5)	23.9 (22.0–25.8)	0.004
Decreased (<21.0)	53 (24%)	18 (32%)	17 (40%)	18 (15%)	0.001
**Coagulation function**					
D-dimer (mg/L)	0.44 (0.22–0.97)	0.78 (0.34–1.38)	0.55 (0.22–1.31)	0.33 (0.22–0.59)	<0.001
Increased (≥0.5)	86 (28%)	40 (48%)	17 (27%)	29 (17%)	<0.001
Prothrombin time (second)	12.9 (12.0–13.8)	12.7 (12.0–14.4)	12.8 (11.7–14.4)	12.9 (12.3–13.6)	0.99
Activated partial thromboplastin time (second)	36.8 (32.65–41.1)	35.3 (32.5–41.2)	34.8 (29.7–40.3)	38.0 (35.0–41.4)	0.04
Fibrinogen (g/L)	3.92 (3.05–4.93)	4.34 (3.30–5.24)	4.43 (3.44–5.48)	3.42 (2.82–4.47)	<0.001
Increased (>4.0)	103 (48%)	34 (52%)	32 (68%)	37 (37%)	0.004
Decreased (<2.0)	9 (4%)	4 (6%)	1 (2%)	4 (4%)	
**Urine routine**					
Positive protein	44 (28%)	17 (40%)	13 (37%)	14 (17%)	0.009
Positive ketones	28 (17%)	11 (24%)	5 (13%)	12 (15%)	0.31
**Inflammatory markers**					
C-reactive protein (mg/L)	17.6 (3.8–41.3)	28.1 (13.0–76.7)	34.9 (8.1–67.8)	8.0 (3.1–30.2)	<0.001
Increased (>8.0)	166 (65%)	61 (85%)	39 (78%)	66 (50%)	<0.001
Erythrocyte sedimentation rate (mm/h)	21 (9-38)	31 (10-59)	25 (12-40)	16 (8-33)	0.03
Procalcitonin (μg/L)	0.10 (0.06–0.16)	0.13 (0.07–0.30)	0.10 (0.09–0.25)	0.08 (0.04–0.10)	<0.001
Increased (>0.5)	11 (8%)	7 (13%)	3 (10%)	1 (2%)	0.002
Lactate dehydrogenase (g/L)	225 (180–291)	264 (225–373)	258 (198–336)	195 (167–244)	<0.001
Increased (>245.0)	99 (41%)	41 (64%)	28 (51%)	30 (25%)	<0.001
Decreased (<109.0)	1 (0.4%)	0 (0%)	0 (0%)	1 (0.8%)	
Neutrophil-to-lymphocyte ratio	2.65 (1.73–4.95)	4.07 (2.20–7.68)	3.00 (1.74–4.75)	2.19 (1.50–3.46)	<0.001
Platelet-to-lymphocyte ratio	163 (116–236)	177 (136–295)	154 (100–229)	156 (117–219)	0.08
**Metabolic variables**					
BMI (kg/m^2^)	23.5 (21.1–25.2)	23.6 (22.7–26.1)	24.2 (21.2–25.5)	22.8 (20.8–24.9)	0.21
Fasting plasma glucose (mmol/L)	5.62 (5.07–6.88)	8.35 (6.91–11.69)	6.11 (5.82–6.54)	5.04 (4.72–5.26)	<0.001
Total cholesterol (mmol/L)	3.68 (3.27–4.47)	3.57 (3.13–4.00)	3.74 (3.30–4.38)	3.80 (3.41–4.60)	0.08
Triglyceride (mmol/L)	1.11 (0.84–1.53)	1.29 (0.85–1.68)	1.14 (0.86–1.49)	1.05 (0.82–1.44)	0.10
HDL-C (mmol/L)	1.12 (0.89–1.37)	1.03 (0.87–1.32)	1.02 (0.89–1.37)	1.17 (0.94–1.43)	0.09
LDL-C (mmol/L)	2.19 (1.77–2.72)	1.99 (1.60–2.56)	2.21 (1.79–2.71)	2.24 (1.91–2.88)	0.054
Uric acid (μmol/L)	250 (192–328)	265 (186–328)	252 (203–337)	244 (192–321)	0.61
Systolic blood pressure (mmHg)	125 (119–134)	130 (120–142)	126 (120–133)	123 (118–130)	0.003
Diastolic blood pressure (mmHg)	76 (70–83)	77 (70–85)	78 (70–87)	75 (70–82)	0.44

*Data are shown as median (IQR) and n (%)*.

*P-values were derived from χ^2^-test, Fisher's exact test, or Kruskal Wallis test when appropriate*.

*COVID-19, 2019 novel coronavirus; IFG, impaired fasting glucose; NFG, normal fasting glucose; eGFR, estimated glomerular filtration rate; CKMB, creatine kinase MB; HDL-C, high density lipoprotein cholesterol; LDL-C, low density lipoprotein cholesterol*.

Compared with those with NFG, patients with IFG and diabetes were more likely to have abnormal levels of laboratory markers, including increased liver enzymes, decreased albumin and estimated glomerular filtration rate (eGFR), elevated cystatin C, creatine kinase, d-dimer and fibrinogen, positive urine protein, higher levels of inflammatory markers (CRP, ESR, procalcitonin, LDH, and neutrophil-to-lymphocyte ratio) and systolic blood pressure (Table 2). No significant differences were found in BMI, lipid profiles, uric acid and diastolic blood pressure across the groups (Table 2).

Patient with diabetes and IFG were more frequently infected with bacteria than those with NFG (11, 8, and 1%, respectively; Supplementary Table 3). No significant differences were found in the chest CT findings among the three groups (Supplementary Table 3).

### Clinical Severity, Complications, and Treatment Regimens

The proportion of severe cases was higher in patients with diabetes (36 [43%]) and IFG (20 [32%]), compared with those with NFG (19 [11%]), and the corresponding odds ratio (OR) and 95% confidence interval (CI) was 4.04 (1.87–8.75) for diabetes and 2.86 (1.19–6.83) for IFG compared to NFG (Supplementary Table 4). The association remained significant even after adjustment for laboratory markers.

Compared with patients with NFG, patients with IFG and diabetes were more likely to develop ARDS (3 [2%], 2 [3%], and 7 [8%]), acute kidney injury (0 [0%], 1 [2%], and 4 [5%]), and septic shock (3 [2%], 5 [8%], and 15 [18%]). Among patients with diabetes, 2 had diabetic ketoacidosis and 1 had drug-induced hypoglycemic coma during follow-up, while none developed hyperosmolar hyperglycemic state (Table 3).

**Table 3:** Complications and treatment regimens of patients with COVID-19 according to diabetes status.

	**All patients (*n* = 312)**	**Diabetes (*n* = 84)**	**IFG (*n* = 62)**	**NFG (*n* = 166)**	***P*-value**
**Any complications**	47 (15%)	23 (27%)	11 (17%)	13 (8%)	<0.001
ARDS	12 (4%)	7 (8%)	2 (3%)	3 (2%)	0.04
Acute kidney injury	5 (2.0%)	4 (5%)	1 (2%)	0 (0%)	0.01
Cardiac injury	18 (6%)	7 (8%)	3 (5%)	8 (5%)	0.49
Rhabdomyolysis	1 (0.3%)	0 (0%)	1 (2%)	0 (0%)	0.20
Septic shock	23 (7%)	15 (18%)	5 (8%)	3 (2%)	<0.001
Diabetic ketoacidosis	2 (1%)	2 (2%)	0 (0%)	0 (0%)	0.11
Hyperosmolar hyperglycemic state	0 (0%)	0 (0%)	0 (0%)	0 (0%)	NA
Hypoglycemic coma	1 (0.3%)	1 (1%)	0 (0%)	0 (0%)	0.47
**Treatment**					
Oxygen therapy	231 (74%)	70 (83%)	53 (85%)	108 (65%)	<0.001
Nasal tube	190 (61%)	45 (54%)	43 (69%)	102 (61%)	<0.001
High-flow nasal cannula	9 (3%)	4 (5%)	4 (6%)	1 (1%)	
Non-invasive ventilator	25 (8%)	18 (21%)	4 (6%)	3 (2%)	
Invasive ventilator	7 (2%)	3 (4%)	2 (3%)	2 (1%)	
**Antiviral treatment**					
Oseltamivir	186 (60%)	52 (62%)	33 (53%)	101 (61%)	0.51
Ganciclovir	98 (31%)	34 (40%)	25 (40%)	39 (23%)	0.006
Lopinavir and ritonavir	39 (13%)	13 (15%)	8 (13%)	18 (11%)	0.58
Arbidol hydrochloride	227 (73%)	52 (62%)	45 (73%)	130 (78%)	0.02
Ribavirin	46 (15%)	11 (13%)	13 (21%)	22 (13%)	0.30
Interferon	140 (45%)	32 (38%)	28 (45%)	80 (48%)	0.32
>2 antiviral agents	138 (44%)	34 (40%)	31 (50%)	73 (44%)	0.52
**Antibacterial treatment**					
Intravenous antibiotics	242 (78%)	74 (88%)	52 (84%)	116 (70%)	0.002
Numbers of antibiotics	3 (2-4)	3 (2-4)	3 (2-4)	3 (2-4)	0.55
>2 antibacterial agents	34 (11%)	15 (18%)	5 (8%)	14 (8%)	0.06
Antifungal treatment	22 (7%)	9 (11%)	6 (10%)	7 (4%)	0.08
Glucocorticoids	129 (41%)	43 (51%)	36 (59%)	50 (30%)	<0.001
Intravenous immunoglobulin therapy	131 (42%)	47 (56%)	31 (51%)	53 (32%)	<0.001
Thymosin	92 (30%)	24 (29%)	24 (39%)	44 (27%)	0.17

*Data are shown as median (IQR) and n (%)*.

*P-values were derived from χ^2^-test, Fisher's exact test, or Kruskal Wallis test when appropriate*.

*COVID-19, 2019 novel coronavirus; IFG, impaired fasting glucose; NFG, normal fasting glucose; ARDS, acute respiratory distress syndrome coronavirus*.

The treatment regimens are shown in the Table 3. Patients with diabetes and IFG, compared with patients with NFG, were more likely to receive the treatment of ganciclovir (34 [40%], 25 [40%] vs. 39 [23%]), intravenous antibacterial agents (74 [88%], 52 [84%] vs. 116 [70%]), glucocorticoids (43 [51%], 36 [59%] vs. 50 [30%]), and intravenous immunoglobulin therapy (47 [56%], 31 [51%] vs. 53 [32%]), while were less likely to be treated with arbidol hydrochloride (52 [62%], 45 [73%] vs. 130 [78%]; Table 3).

### Primary Endpoints and Mortality of the Patients

Primary composite endpoints occurred in 46 (15%) patients, including 32 (10%) who underwent mechanical ventilation, 9 (3%) who were admitted to the ICU, and 33 (11%) who died. Among the 33 deaths, majority were respiratory failure (27 [81.8%]), and the rest were multiorgan failure (5 [15.2%]) and cardiovascular event (1 [3.0%]; Supplementary Table 5).

Compared with patients with NFG, patients with IFG and diabetes were more likely to develop primary composite events (9 [5%], 11 [18%], and 26 [31%]; Supplementary Table 5), including more receiving mechanical ventilation (5 [3%], 6 [10%], and 21 [25%]), more deaths (4 [2%], 9 [15%], and 20 [24%]), but similar admission rate to ICU (3 [2%], 2 [3%], and 4 [5%]).

**Figure 1:**
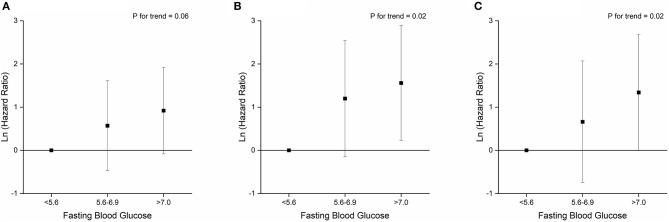
Associations between fasting blood glucose levels and primary composite endpoint **(A)**, mortality **(B)**, and mechanical ventilation **(C)**.

Patients with IFG and diabetes had significantly escalated risks of reaching to the composite endpoint or death compared with those with NFG (all *P* < 0.05; Supplementary Figure 1). After adjustment for age, sex, hospitals and other comorbidities, diabetes remained as a significant predictor for the composite endpoints (HR 3.53, 95% CI 1.48–8.40; Table 4), while the association with IFG was not statistically significant (HR 1.42, 95% CI 0.53–3.81). Per-SD increment of fasting glucose levels was associated with 25% (2–53%) higher risk of composite endpoints. Both IFG and diabetes were associated with higher risk of mortality among patients with COVID-19, and the HR (95% CI) was 4.11 (1.15–14.74) and 6.25 (1.91–20.45), respectively, and per-SD increment of fasting glucose levels was associated with 31% (4–65%) higher risk of mortality (Table 4; Figure 1).

**Table 4:** Association between diabetes status and risk of adverse outcomes among patients with COVID-19.

	**Diabetes**	**IFG**	**NFG**	***P*-value for trend**	**Per SD increase in plasma fasting glucose levels**
**Primary composite outcomes**					
Cases/person-days	26/1,875	11/1,455	9/4,419		
Model 1	2.76 (1.22–6.24)	1.61 (0.63–4.11)	1.00	0.001	1.20 (1.00–1.44)
Model 2	3.53 (1.48–8.40)	1.42 (0.53–3.81)	1.00	0.002	1.25 (1.02–1.53)
Model 3	2.18 (0.89–5.31)	1.21 (0.43–3.39)	1.00	0.045	1.21 (0.98–1.50)
**Mortality**					
Cases/person-days	20/2,059	9/1,516	4/4,521		
Model 1	6.36 (2.10–19.30)	4.02 (1.18–13.64)	1.00	0.001	1.31 (1.07–1.60)
Model 2	6.25 (1.91–20.45)	4.11 (1.15–14.74)	1.00	0.002	1.31 (1.04–1.65)
Model 3	6.87 (1.92–24.58)	4.06 (1.00–16.42)	1.00	0.002	1.37 (1.05–1.79)
**Mechanical ventilation**					
Cases/person-days	21/1,868	6/1,453	5/4,415		
Model 1	3.21 (1.12–9.23)	1.34 (0.39–4.69)	1.00	0.01	1.19 (0.95–1.50)
Model 2	6.33 (1.87–21.48)	1.66 (0.42–6.54)	1.00	0.001	1.22 (0.96–1.55)
Model 3	2.31 (0.76–7.03)	0.95 (0.25–3.66)	1.00	0.047	1.32 (1.01–1.74)

*Model 1, adjusted for age, sex, and hospital*.

*Model 2, adjusted for age, sex, hospital, and comorbidities*.

*Model 3, adjusted for age, sex, hospital, aspartate aminotransferase, estimated glomerular filtration rate, prothrombin time, and procalcitonin*.

In the model adjusted for age, sex, and hospital, the following laboratory variables were found to be associated with increased risks of the primary endpoints: decreased eGFR, increased aspartate aminotransferase (AST), cystatin C, creatine kinase MB (CKMB), prothrombin time (PT), procalcitonin, LDH, and neutrophil-to-lymphocyte ratio (Supplementary Table 6). We selected decreased eGFR, increased AST, PT, and procalcitonin in the final multivariate model, and found that further adjustment for those variables did not materially alter the associations between diabetes and mortality, whereas the associations between diabetes and composite outcome were attenuated to insignificance, as well as the association between IFG and mortality (Model 3, Table 4).

In the sensitivity analysis of excluding the new-diagnosed diabetes (Supplementary Table 7), the above associations remained unchanged.

We further compared the anti-diabetic drugs among patients with diabetes stratified by the status of primary endpoints. Among the 26 patients with diabetes and experienced the primary endpoints, 1 patient used only metformin, while 19 out of 58 patients with diabetes and who did not experience the primary endpoints used metformin (4 vs. 33%, *P* = 0.004; Supplementary Table 8). No dramatic differences were observed for other anti-diabetic drugs between the two groups.

## Discussions

In this multicenter retrospective cohort study among 312 patients with COVID-19 from Wuhan, China, we found that diabetes and IFG were associated with higher risks of primary adverse endpoints and mortality. In addition, dose-response association was also found between fasting plasma glucose levels on hospital admission and risk of adverse prognosis for patients with COVID-19. The associations were independent of other comorbidities, but the association between diabetes and IFG and primary endpoints was partially explained by some laboratory markers.

A number of studies have reported that diabetes was a risk factor for severity and poor prognosis of influenza and other pneumonia diseases. For example, a study among 239 patients with influenza A in Canada reported that diabetes tripled the risk of hospitalization and quadrupled the risk of ICU admission once hospitalized (9); a study among 144 patients with SARS in Canada reported that diabetes was independently associated with poor primary endpoints (death, ICU admission or mechanical ventilation) (26). In another retrospective analysis of 520 patients with SARS in Hong Kong, a known history of diabetes was associated with 3-fold risk of mortality and fasting plasma glucose levels were negatively associated with mortality and hypoxia (10). The risk of developing severe or lethal disease following MERS-CoV infection was increased by 2.47 to 7.24-folds when the patient had comorbid diabetes (11, 12). However, it is still controversial whether diabetes is a major risk factor for severity and poor prognosis of COVID-19 (13, 14). And no study emphasizes on the relationship between prediabetes and severity of COVID-19 until now.

In our study, we observed that patients with hyperglycemia were more likely to be severe cases at the time of hospital admission with an odds ratio of 3.14 for diabetes and 2.36 for IFG compared to NFG. This is consistent with a recent meta-analysis of 31 reports with varied sample sizes (ranged from 21 to 1099) which found that patients with diabetes had a significantly increased odds (OR 2.61; 95% CI 2.05–3.33) of developing severe COVID-19 compared with patients free of diabetes (15). Among 1,590 patients with COVID-19 across China, severe cases were found in 34.6% of patients with diabetes (45/130) and 14.3% of patients without diabetes (209/1,460) (4). However, many of the studies were either preprint reports or did not control for important confounders, such as age, sex and other comorbidities.

A few studies have evaluated the association between comorbid diabetes and poor prognosis among patients with COVID-19. The study by Guan et al. (4) reported that diabetes was associated with a higher risk of composite endpoints (HR 1.59; 95% CI 1.03–2.45) in 1,590 patients with COVID-19 across China; Zhou et al. (27) reported that diabetes was associated with a higher odds of in-hospital death (OR 2.85; 95% CI 1.35–6.05) in the univariable analysis of 191 patients with COVID-19 from two hospitals in Wuhan; a large-scale report among 44,672 confirmed cases of COVID-19 across China found that the crude case fatality rate was 7.3% among patients with diabetes compared to 2.3% in the total samples (28); another research among 7,337 cases with COVID-19 reported recently found that subjects with type 2 diabetes required more medical interventions and had a significantly higher mortality (7.8 vs. 2.7%) than the non-diabetic individuals (20). Therefore, our results are largely consistent with those studies but with additional advantages: we adjusted for some confounding factors, including age, sex, hospital, and comorbidities, and also showed that diabetes and IFG was risk factors for in-hospital death.

Although the relationship between diabetes and COVID-19 has been currently extensively explored, less research has paid attention to the relationship between IFG and worse COVID-19 prognosis. Whether diabetes patients need a rigid glucose control when affected with COVID-19 is still unknown. Previous clinical trials examining the effects of glucose control on mortality have yielded conflicting results (20, 21, 29). Zhu et al. reported well-controlled blood glucose (3.9–10.0 mmol/L) was associated with markedly lower mortality compared to individuals with poorly controlled BG (>10.0 mmol/L) (20). Plasma glucose concentrations with 4–8 mmol/L was recommended as therapeutic aim in the practical recommendations for the management of diabetes patients with COVID-19 (30). In our study, even mild elevated fasting glucose level (5.6–6.9 mmol/L) was associated with increased in-hospital death.

Two small studies (a total of 102 and 138 patients, and 11 and 14 patients had diabetes, respectively) described that patients admitted to ICU had higher prevalence of diabetes compared to those who did not receive ICU care (22, 31); Wu et al. (32) reported that the prevalence of diabetes was higher among patients who developed ARDS (16/84) compared to those with ARDS (6/117) among 201 patients from a hospital in Wuhan. In our study, 9 patients received ICU care, 4 (5%) in patients with diabetes, 2 (3%) in patients with IFG and 3 (2%) in patients with NFG; only 12 patients developed ARDS, 7 [8%] in patients with diabetes, 2 [3%] in patients with IFG and 3 (2%) in patients with NFG. Furthermore, acute kidney injury (5, 2, and 0%) and septic shock (18, 8, and 2%) were also higher in patients with diabetes and IFG compared to patients with NFG. However, we did not perform multivariate analysis because of limited sample size.

Several factors may contribute to the increased risks of severity and poor prognosis of COVID-19 related to hyperglycemia. First, patients with diabetes were more likely to have other comorbidities, such as hypertension, cardiovascular disease, and kidney injuries, and studies have demonstrated that multiple comorbidities increased risk of composite endpoints and mortality among patients with COVID-19 (4, 6, 15, 33, 34). However, we have adjusted for a number of comorbidities in the multivariate models and the associations with diabetes remained significant. Second, patients with diabetes and IFG had a number of worse laboratory markers, including dysregulated immune response, increased proinflammatory reactions, metabolic abnormalities, procoagulant, or impaired fibrinolytic states and which could be related to adverse prognosis for patients with COVID-19 (2, 22, 23, 27, 35). When adjusted for several markers, the association between diabetes and primary endpoints were no long statistically significant, which indicated that the association might be partly explained by those factors, but sample size could also be a factor for the insignificant results. The association between diabetes and mortality was not altered. Third, individuals with diabetes and IFG were at increased risk for bacterial co-infection and septic shock in our study, and bacterial infections have been found to be related to higher risk of mortality in patients with COVID-19 (36).

To the best of our knowledge, our study is the first to classify glycemic status into three different groups (NFG, IFG, and diabetes) and also investigate the relations between fasting glucose levels at admission and adverse prognosis among patients with COVID-19. We have controlled for a number of covariates and tried to evaluate the laboratory markers in the pathway of the associations. Nevertheless, out study also has several limitations. First, we did not know the exact type of diabetes and assumed that majority, if not all, should be type 2 diabetes. Second, HbA1c was not routinely measured at the time of hospital admission, particularly for those without diabetes, therefore, we could not evaluate the association between HbA1c and risk of adverse outcomes because of lack of data. Third, we did not have information on some confounding factors such as socioeconomic status and lifestyle factors, and residual confounding was possible as in any observational studies. Besides, only 30% (93 out of 312) of the participants had data available on BMI, and thus we could not adjust for BMI in the multivariable models. Fourth, although we have retrieved medication information, we still could not evaluate the impact of different types or combinations of anti-diabetic drugs on the prognosis, which would require a much larger sample size. Fifth, we did not have information on glycemic control measures, kinetics of viral load and antibody titers during the follow-up, and more studies are still needed to understand the impact of those factors in the development of adverse outcomes. Finally, our study had limited sample size and all patients were Chinese recruited from 5 hospitals in Wuhan city by convenience sampling; furthermore, the prevalence rates of diabetes and IFG were higher than the rates in the general population or in patients with COVID-19 from national surveys of Chinese. However, representativeness was not strictly required in cohort studies to generalize the observed associations (37). Nevertheless, studies in other populations are needed to further validate our findings.

## Conclusions

In this retrospective cohort of 312 Chinese patients with COVID-19, we found that diabetes was associated with higher risks of composite adverse endpoints (mechanical ventilation, admission to ICU, or death) and mortality, and IFG was also associated with higher risk of mortality. The associations appeared to be dose-dependent and were not explained by other comorbidities. Given the global high prevalence of diabetes in adults and the current COVID-19 pandemic, many patients with diabetes are infected with SARS-COV-2; therefore, the knowledge provided in this study could be useful for understanding the clinical characteristics of patients with diabetes and COVID-19, and to help develop more targeted and effective management strategies for those patients to reduce the poor prognosis. More studies are still warranted to validate our findings and further understand the potential mechanisms.

## Data Availability Statement

The raw data supporting the conclusions of this article will be made available by the authors, without undue reservation.

## Ethics Statement

The studies involving human participants were reviewed and approved by the Union Hospital, Tongji Medical College, Huazhong University of Science and Technology. Written informed consent for participation was not required for this study in accordance with the national legislation and the institutional requirements.

## Author Contributions

JZha, WK, TZ, LC, and AP co-conceived the study. YX, LL, QL, LY, QW, HanyW, GL, XZ, KQ, YLi, HanW, YW, XS, HLiu, SX, YLiu, and ZC collected the epidemiological and clinical data. HanyW, KQ, HanW, YW, GL, and XZ summarized all data. PX did the analysis. JZha, WK, KQ, and YW drafted the manuscript. HLi, JZhe, HS, WX, and YH contributed to the discussion. LC, AP, and TZ revised the final manuscript. TZ was guarantor of this work and, as such, had full access to all the data in the study and takes responsibility for the integrity of the data and the accuracy of the data analysis. All authors contributed to the article and approved the submitted version.

## Conflict of Interest

The authors declare that the research was conducted in the absence of any commercial or financial relationships that could be construed as a potential conflict of interest.
