# Supplementary material for: Impaired Fasting Glucose and Diabetes Are Related to Higher Risks of Complications and Mortality Among Patients With Coronavirus Disease 2019
Source: Front Endocrinol (Lausanne). 2020 Jul 10;11:525. doi: 10.3389/fendo.2020.00525 (PMC7365851; doi:10.3389/fendo.2020.00525)
Supplement: Supplementary file 1 [file Data_Sheet_1.docx]

**Supplementary material**

**METHODS**

**Definitions of COVID-19 diagnosis**

Case definition for COVID-19 was divided into suspected case and confirmed case based on the epidemiological history, clinical features and laboratory examination by the National Health Commission of the People’s Republic of China (1). Only confirmed cases were analyzed in our study.

**Supplementary Table 1：Case Definition for Surveillance of Coronavirus Disease 2019 (COVID-19) by Chinese Health Commission**

| Suspected case  Present at least two of the following conditions of  i. Fever and/or respiratory symptoms (eg, cough, myalgia, fatigue)  ii. Imaging features of viral pneumonia  iii. Normal or low white blood cell count or reduced lymphocyte in early onset  AND  One or more of the following exposures during the 14 days prior to onset of symptoms  1). Travel or residence history in Wuhan, other areas with recent local transmission of COVID-19, or the local community with confirmed patient  2). Close contact* with patient with laboratory-confirmed COVID-19 (positive for nucleic acid test)  3). Close contact with people from Wuhan or surrounding areas or local communities with fever or respiratory symptoms case report  4). Cluster onset (two or more cases of fever and/or respiratory symptoms occurred within 2 weeks in a small area such as home, office, school and class).  Patients without exposure history should meet all of conditions i, ii and iii. |
| --- |
| **Clinically diagnosed case (added in the trial fifth edition but deleted in the trial sixth and seventh edition)**  The suspected case with typical imaging findings of pneumonia (only for Hubei) |
| Confirmed case  Suspected cases have at least one of the following etiological evidence  i. Positive real-time reverse-transcriptase polymerase chain reaction of the patient’s respiratory or blood specimen for COVID-19 nucleic acid  ii. Viral gene sequences in respiratory or blood specimen are highly homologous to COVID-19  iii. Positive serum specific IgM and IgG antibody for COVID-19; The serum specific IgG antibody for COVID-19 changed from negative to positive or the recovery period was 4 times or more higher than the acute period. |

*Close contact is defined as healthcare-related exposures, including direct care for patients with confirmed COVID-19, collaboration with healthcare workers with confirmed COVID-19, visiting or staying in the same closed environment with patients with confirmed COVID-19, or members who live in the same family environment with patients with confirmed COVID-19.

**Definitions of non-severe or severe types of pneumonia**

SARS-COV-2 pneumonia was divided into non-severe or severe types according to the Infectious Diseases Society of America/American Thoracic Society CAP severity criteria that have been validated (2).Severe type was deﬁned as present in patients with either one major criterion or three or more minor criteria, otherwise it was defined as non-severe type.

**Supplementary Table 2: 2007 Infectious Diseases Society of America/American Thoracic Society Criteria for Deﬁning Severe Community-acquired Pneumonia**

**Validated deﬁnition includes either one major criterion or three or more minor criteria**

**Minor criteria**

Respiratory rate≥30 breaths/min

PaO2/FIO2 ratio≤250

Multilobar inﬁltrates

Confusion/disorientation

Uremia (blood urea nitrogen level≥20 mg/dl)

Leukopenia* (white blood cell count<4,000 cells/ml)

Thrombocytopenia (platelet count<100,000/μl)

Hypothermia (core temperature<36℃)

Hypotension requiring aggressive ﬂuid resuscitation

**Major criteria**

Septic shock with need for vasopressors

Respiratory failure requiring mechanical ventilation

*Due to infection alone (i.e., not chemotherapy induced).

**Definitions of diabetes and clinical complications**

Diabetes was diagnosed according to the criteria of the 2014 guidelines of the American Diabetes Association (3). Anyone with a previous diagnosis of diabetes, or anti-diabetic medication use or fasting plasma glucose (FPG) ≥ 7.0 mmol/l at admission was defined as diabeties. Impaired fasting glucose (IFG) was defined as glucose levels between 5.6 and 6.9 mmol/L.

Acute respiratory distress syndrome (ARDS) and septic shock were defined according to the WHO interim guidance (4).

Acute kidney injury was defined based on the highest serum creatinine level and urine output. Specifically, the diagnosis could be made based on any of the following criteria: an increase in serum creatinine levels by 0.3 mg/dl or greater (26.5 µmol/l or greater) within 48 hours; an increase in serum creatinine levels up to 1.5 times of the baseline level or greater, which was known or presumed to have occurred within 7 days; an urine volume below 0.5 ml/kg/h for 6 consecutive hours (5).

Co-infection was diagnosed by the occurrence of hospital-acquired pneumonia or bacteremia, plus a positive culture of a new pathogen from the blood or lower respiratory tract specimen (including sputum, bronchoalveolar lavage fluid or tracheal aspirates) obtained at least 8 hours after admission (6).

Cardiac injury was defined as one or more of the following: blood level of cardiac biomarkers (TNI or CK-MB) above the 99th percentile upper reference limit, new abnormalities in electrocardiography, including supraventricular tachycardia, ventricular tachycardia, atrial fibrillation, ventricular fibrillation, bundle branch block, ST-segment elevation/depression, T-wave ﬂattening/inversion, and QT interval prolongation, new abnormalities in echocardiography, including decreased EF value (EF < 50%) or a worsening of the underlying state (patients with basal state of EF <50%), regional/global ventricular wall motion abnormalities, the presence of pericardial effusion, and pulmonary arterial hypertension (PAH) (7).

Rhabdomyolysis was defined if the muscle pain or muscle weakness took place on admission and the creatine kinase level was greater than 10 times the upper limit of normal (8).

Diabetic ketoacidosis (DKA) was diagnosed according to the criteria of the American Diabetes Association (ADA) consensus guidelines (2009). It suggests using a glucose threshold of > 250 mg/dL (13.9 mmol/L), presence of positive serum and urine ketones with an anion gap, and arterial pH < 7.3 to make the diagnosis of DKA. Anion gap is calculated with the formula = sodium [Na^+^] – (chloride [Cl^-^] + [HCO3^−^] (9).

The diagnostic criteria for Hyperglycemic Hyperosmolar State (HHS) includes a plasma glucose of over 600 mg/dl, and effective osmolality> 320 mOsm/kg, and the absence of ketoacidosis. Effective osmolality is calculated with the formula = 2[measured Na^+^ (mEq/L)] + glucose (mg/dL)/18 (9).

Hypoglycemic coma was defined as a state in which the patient was not arousable (or responded only to pain), with a blood glucose concentration of 2.72 mmol/L (49mg/dL) or less, and responded symptomatically (a return of consciousness) to the administration of intravenous glucose (10).

**Supplementary Table 3. Co-infection and radiologic findings of patients with** **COVID-19 according to diabetes status**

|  | **All Patients (n=312)** | **Diabetes (n=84)** | **IFG (n=62)** | **NFG (n=166)** | ***P* value** |
| --- | --- | --- | --- | --- | --- |
| **Co-infection** |  |  |  |  |  |
| Any co-infection | 30 (10%) | 11 (13%) | 4 (6%) | 15 (9%) | 0.38 |
| Mycoplasma | 16 (9%) | 7 (12%) | 0 (0%) | 9 (10%) | 0.06 |
| Chlamydia | 4 (2%) | 3 (5%) | 0 (0%) | 1 (1%) | 0.20 |
| Other respiratory viruses | 17 (5%) | 6 (7%) | 4 (6%) | 7 (4%) | 0.53 |
| Bacteria | 16 (5%) | 9 (11%) | 5 (8%) | 2 (1%) | 0.001 |
| Fungus | 10 (3%) | 4 (5%) | 3 (5%) | 3 (2%) | 0.27 |
| **Chest CT findings** |  |  |  |  |  |
| Number of lung lobes involving | 2 (1-3) | 2 (2-3) | 2 (1-2) | 2 (1-3) | 0.18 |
| Unilateral pneumonia | 39 (13%) | 6 (8%) | 9 (15%) | 24 (15%) | 0.28 |
| Bilateral pneumonia | 255 (87%) | 70 (92%) | 51 (85%) | 134 (85%) |  |
| CT features |  |  |  |  |  |
| Multiple mottling | 203 (69%) | 55 (73%) | 39 (66%) | 109 (69%) | 0.64 |
| Ground -glass opacity | 148 (51%) | 31 (41%) | 27 (46%) | 90 (57%) | 0.06 |
| Consolidation | 37 (13%) | 7 (9%) | 8 (14%) | 22 (14%) | 0.59 |
| Change of CT features |  |  |  |  |  |
| Alleviated | 169 (73%) | 32 (65%) | 28 (62%) | 109 (80%) | 0.01 |
| Aggravated | 42 (18%) | 15 (31%) | 11 (24%) | 16 (12%) |  |

Data are shown as median (IQR) and n (%).

*P* values were derived from χ² test, Fisher’s exact test, or Mann-Whitney U test when appropriate.

COVID-19, 2019 novel coronavirus; IFG, impaired fasting glucose; NFG, normal fasting glucose.

**Supplementary Table 4. Association between diabetes status and risk of severity status of patients with COVID-19**

|  | **NFG (n=166)** | **IFG (n=62)** | **Diabetes (n=84)** | ***P* value for trend** |
| --- | --- | --- | --- | --- |
| Cases (%) | 19 (11%) | 20 (32%) | 36 (43%) |  |
| Model 1 | 1.00 | 2.36 (1.06-5.28) | 3.14 (1.54-6.39) | 0.002 |
| Model 2 | 1.00 | 2.86 (1.19-6.83) | 4.04 (1.87-8.75) | <0.001 |
| Model 3 | 1.00 | 2.13 (0.87-5.23) | 2.57 (1.17-5.65) | 0.02 |

Model 1, adjusted for age, sex and hospital.

Model 2, adjusted for age, sex, hospital and comorbidities.

Model 3, adjusted for age, sex, hospital, aspartate aminotransferase, estimated glomerular filtration rate, prothrombin time and procalcitonin.

Missing values were treated as missing indicators in the regression models.

**Supplementary Table 5. Clinical outcomes and causes of death of patients with COVID-19 according to diabetes status**

|  | **All Patients (n=312)** | **Diabetes (n=84)** | **IFG (n=62)** | **NFG (n=166)** | ***P* value** |
| --- | --- | --- | --- | --- | --- |
| **Clinical outcome** |  |  |  |  |  |
| Primary composite end-point event | 46 (15%) | 26 (31%) | 11 (18%) | 9 (5%) | <0.001 |
| Admission to ICU | 9 (3%) | 4 (5%) | 2 (3%) | 3 (2%) | 0.39 |
| Days from illness onset to transfer to ICU | 7 (5-10) | 4 (2.5-10) | 6.5 (6-7) | 10 (8-13) | 0.29 |
| Mortality | 33 (11%) | 20 (24%) | 9 (15%) | 4 (2%) | <0.001 |
| Days from illness onset to death | 17 (13-23) | 17 (12-21.5) | 15 (13-25) | 19 (16-22) | 0.77 |
| Days in hospital (dead patients excluded) | 17 (12-22) | 18 (13-22) | 15 (11-20) | 16 (11-22) | 0.52 |
| **Cause of death** |  |  |  |  |  |
| Respiratory failure | 27 (9%) | 16 (19%) | 7 (11%) | 4 (2%) | <0.001 |
| Multiorgan failure | 5 (2%) | 3 (4%) | 2 (3%) | 0 (0%) |  |
| Cardiovascular incidents | 1 (0.3%) | 1 (1%) | 0 (0%) | 0 (0%) |  |
| Diabetic crisis | 0 (0%) | 0 (0%) | 0 (0%) | 0 (0%) |  |
| Upper gastrointestinal hemorrhage | 0 (0%) | 0 (0%) | 0 (0%) | 0 (0%) |  |
| Other diseases | 0 (0%) | 0 (0%) | 0 (0%) | 0 (0%) |  |

Primary composite end-point event contains admission to ICU, or mechanical ventilation, or death.

Data are shown as median (IQR) and n (%).

*P* values were derived from χ² test, Fisher’s exact test, or Mann-Whitney U test when appropriate.

COVID-19, 2019 novel coronavirus; IFG, impaired fasting glucose; NFG, normal fasting glucose; ICU, intensive care unit.

**Supplementary Table 6. Association between levels of biomarkers and adverse outcomes among patients with COVID-19**

|  | **Primary composite outcomes** | | **Mortality** | | **Mechanical ventilation** | |
| --- | --- | --- | --- | --- | --- | --- |
|  | **HR (95% CI)** | ***P* value** | **HR (95% CI)** | ***P* value** | **HR (95% CI)** | ***P* value** |
| **Blood routine** |  |  |  |  |  |  |
| Neutrophils (×10^9^ per L) |  |  |  |  |  |  |
| Increased (>6.3) | 2.02 (0.96-4.24) | 0.06 | 3.15 (1.33-7.46) | 0.01 | 2.79 (1.15-6.74) | 0.02 |
| Decreased (<1.8) | 0.49 (0.11-2.07) | 0.33 | 0.71 (0.16-3.10) | 0.65 | 1.07 (0.24-4.80) | 0.93 |
| Lymphocytes (×10^9^ per L) |  |  |  |  |  |  |
| Decreased (<1.1) | 1.73 (0.77-3.85) | 0.18 | 2.70 (0.98-7.45) | 0.06 | 1.76 (0.70-4.44) | 0.23 |
| Eosinophils (×10^9^ per L) |  |  |  |  |  |  |
| Decreased (<0.02) | 2.27 (1.11-4.61) | 0.02 | 2.40 (1.01-5.70) | 0.05 | 2.65 (1.17-6.00) | 0.02 |
| Basophils (×10^9^ per L) |  |  |  |  |  |  |
| Increased (>0.06) | 1.23 (0.36-4.15) | 0.74 | 1.60 (0.46-5.54) | 0.46 | 1.15 (0.26-5.11) | 0.85 |
| Hemoglobin (g/L) |  |  |  |  |  |  |
| Increased (>150) | 0.92 (0.23-3.61) | 0.91 | 2.54 (0.47-13.64) | 0.28 | 0.49 (0.10-2.45) | 0.38 |
| Decreased (<115) | 0.69 (0.28-1.67) | 0.41 | 1.05 (0.40-2.76) | 0.92 | 0.33 (0.10-1.07) | 0.06 |
| Platelets (×10^9^ per L) |  |  |  |  |  |  |
| Decreased (<120) | 1.83 (0.95-3.53) | 0.07 | 1.53 (0.69-3.39) | 0.29 | 1.56 (0.71-3.43) | 0.27 |
| **Blood biochemistry** |  |  |  |  |  |  |
| Alanine aminotransferase (U/L) |  |  |  |  |  |  |
| Increased (>35.0) | 1.55 (0.78-3.06) | 0.21 | 2.51 (1.15-5.47) | 0.02 | 1.31 (0.57-3.02) | 0.53 |
| Aspartate aminotransferase (U/L) |  |  |  |  |  |  |
| Increased (>40.0) | 2.75 (1.45-5.21) | 0.002 | 4.42 (2.02-9.64) | <0.001 | 3.60 (1.63-7.93) | 0.002 |
| Albumin (g/L) |  |  |  |  |  |  |
| Decreased (<35.0) | 1.19 (0.57-2.49) | 0.64 | 1.38 (0.59-3.22) | 0.45 | 0.95 (0.40-2.28) | 0.91 |
| eGFR (mL/min/1.73m^2^) |  |  |  |  |  |  |
| Decreased (<90) | 3.02 (1.35-6.77) | 0.01 | 3.25 (1.23-8.63) | 0.02 | 2.13 (0.86-5.28) | 0.10 |
| Cystatin C (mg/L) |  |  |  |  |  |  |
| Increased (>1.15) | 3.84 (1.36-10.83) | 0.01 | 3.17 (1.01-9.93) | 0.05 | 3.04 (0.88-10.54) | 0.08 |
| Creatine kinase (U/L) |  |  |  |  |  |  |
| Increased (>140.0) | 2.17 (0.96-4.94) | 0.06 | 2.61 (1.06-6.44) | 0.04 | 2.95 (1.15-7.61) | 0.02 |
| Troponin I (ng/L) |  |  |  |  |  |  |
| Increased (>26.2) | 3.81 (0.65-22.31) | 0.14 | 6.34 (0.62-65.20) | 0.12 | 5.07 (0.84-30.55) | 0.08 |
| CKMB (ng/ml) |  |  |  |  |  |  |
| Increased (≥6.6) | 10.33 (1.65-64.57) | 0.01 | 11.41 (1.59-81.83) | 0.02 | 14.38 (1.26-164.54) | 0.03 |
| Total carbon dioxide (mmol/L) |  |  |  |  |  |  |
| Decreased (<21.0) | 1.84 (0.76-4.43) | 0.18 | 2.49 (0.91-6.78) | 0.08 | 1.29 (0.43-3.87) | 0.65 |
| **Coagulation function** |  |  |  |  |  |  |
| D-dimer (mg/L) |  |  |  |  |  |  |
| Increased (≥0.5) | 2.68 (0.89-8.09) | 0.08 | 4.24 (0.84-21.45) | 0.08 | 2.21 (0.69-7.11) | 0.18 |
| Prothrombin time (second) |  |  |  |  |  |  |
| Increased (>16) | 2.56 (1.15-5.71) | 0.02 | 6.67 (2.57-17.33) | <0.001 | 2.35 (0.90-6.15) | 0.08 |
| Decreased (<11) | 1.28 (0.27-6.07) | 0.76 | 0.75 (0.09-6.40) | 0.79 | 1.92 (0.35-10.61) | 0.45 |
| Activated partial thromboplastin time (second) |  |  |  |  |  |  |
| Increased (>43.5) | 0.58 (0.21-1.54) | 0.27 | 0.26 (0.06-1.06) | 0.06 | 0.53 (0.17-1.61) | 0.26 |
| Decreased (<28.0) | 0.58 (0.18-1.91) | 0.37 | 0.44 (0.09-2.26) | 0.32 | 0.49 (0.12-1.90) | 0.30 |
| Fibrinogen (g/L) |  |  |  |  |  |  |
| Increased (>4.0) | 1.04 (0.45-2.41) | 0.92 | 1.65 (0.60-4.53) | 0.34 | 0.83 (0.33-2.10) | 0.70 |
| Decreased (<2.0) | 0.98 (0.29-3.33) | 0.98 | 2.61 (0.58-11.74) | 0.21 | 0.78 (0.19-3.20) | 0.73 |
| **Urine routine** |  |  |  |  |  |  |
| Positive protein |  |  |  |  |  |  |
| Positive | 2.41 (0.79-7.38) | 0.12 | 22.97 (3.33-158.50) | 0.002 | 1.83 (0.56-6.01) | 0.32 |
| Positive ketones |  |  |  |  |  |  |
| Positive | 1.31 (0.35-4.90) | 0.69 | 3.72 (0.85-16.28) | 0.08 | 0.44 (0.06-3.54) | 0.44 |
| **Inflammatory markers** |  |  |  |  |  |  |
| C-reactive protein (mg/L) |  |  |  |  |  |  |
| Increased (>8.0) | 2.48 (0.53-11.54) | 0.25 | 5.39 (0.66-43.76) | 0.11 | 2.23 (0.47-10.56) | 0.31 |
| Procalcitonin (μg/L) |  |  |  |  |  |  |
| Increased (>0.5) | 3.81 (1.64-8.84) | 0.002 | 8.99 (3.35-24.14) | <0.001 | 3.06 (1.07-8.74) | 0.04 |
| lactate dehydrogenase (g/L) |  |  |  |  |  |  |
| Increased (>245.0) | 3.44 (1.53-7.75) | 0.003 | 6.24 (2.09-18.66) | 0.001 | 5.07 (1.70-15.14) | 0.004 |
| NLR | 1.05 (1.03-1.07) | <0.001 | 1.06 (1.04-1.09) | <0.001 | 1.04 (1.02-1.07) | <0.001 |

Logistic regression models were adjusted for age, sex and hospital.

COVID-19, 2019 novel coronavirus; IFG, impaired fasting glucose; NFG, normal fasting glucose; eGFR, estimated glomerular filtration rate; CKMB, creatine kinase MB; NLR, neutrophil-to-lymphocyte ratio; Primary composite end-point event contains admission to ICU, or mechanical ventilation, or death.

**Supplementary Table 7. Sensitivity analysis of association between diabetes status and risk of adverse outcomes among patients with COVID-19**

|  | **NFG** | **IFG** | **Diabetes** | ***P* value for trend** |
| --- | --- | --- | --- | --- |
| **Primary composite outcomes** |  |  |  |  |
| Cases/person-days | 9/4419 | 11/1455 | 17/1297 |  |
| Model 1 | 1.00 | 1.61 (0.62-4.17) | 2.25 (0.92-5.50) | 0.07 |
| Model 2 | 1.00 | 1.41 (0.52-3.82) | 2.86 (1.11-7.33) | 0.02 |
| Model 3 | 1.00 | 1.06 (0.36-3.11) | 1.51 (0.57-3.98) | 0.33 |
| **Mortality** |  |  |  |  |
| Cases/person-days | 4/4521 | 9/1516 | 13/1406 |  |
| Model 1 | 1.00 | 4.36 (1.27-15.01) | 5.78 (1.80-18.59) | 0.003 |
| Model 2 | 1.00 | 3.79 (1.04-13.86) | 4.90 (1.40-17.20) | 0.01 |
| Model 3 | 1.00 | 4.21 (0.97-18.34) | 6.00 (1.53-23.58) | 0.01 |
| **Mechanical ventilation** |  |  |  |  |
| Cases/person-days | 5/4415 | 6/1453 | 13/1301 |  |
| Model 1 | 1.00 | 1.27 (0.36-4.49) | 2.25 (0.70-7.19) | 0.13 |
| Model 2 | 1.00 | 1.69 (0.42-6.74) | 4.56 (1.20-17.37) | 0.02 |
| Model 3 | 1.00 | 0.70 (0.17-2.94) | 1.35 (0.38-4.76) | 0.40 |

Model 1, adjusted for age, sex and hospital.

Model 2, adjusted for age, sex, hospital and comorbidities.

Model 3, adjusted for age, sex, hospital, aspartate aminotransferase, estimated glomerular filtration rate, prothrombin time and procalcitonin.

Missing values were treated as missing indicators in the regression models.

COVID-19, 2019 novel coronavirus; IFG, impaired fasting glucose; NFG, normal fasting glucose. Primary composite end-point event contains admission to ICU, or mechanical ventilation, or death.

**Supplementary Table 8. Hypoglycemic agents of hospitalized patients who suffered from 2019-nCoV pneumonia according to disease severity and primary composite end-point event**

|  | **All patients (n=84)** | **Disease Severity** | | | **Primary composite end-point event** | | |
| --- | --- | --- | --- | --- | --- | --- | --- |
|  |  | **Non-severe pneumonia (n=48)** | **Severe pneumonia (n=36)** | ***P* value** | **Yes (n=26)** | **No (n=58)** | ***P* value** |
| **Hypoglycemic agents in hospital** |  |  |  |  |  |  |  |
| Insulinotropic agents | 12 (14%) | 7 (15%) | 5 (14%) | 0.93 | 2 (8%) | 10 (17%) | 0.33 |
| Metformin | 20 (24%) | 16 (33%) | 4 (11%) | 0.02 | 1 (4%) | 19 (33%) | 0.004 |
| Thiazolidinediones | 4 (5%) | 3 (6%) | 1 (3%) | 0.63 | 0 | 4 (7%) | 0.31 |
| Alpha-glucosidase inhibitors | 31 (37%) | 19 (40%) | 12 (33%) | 0.56 | 6 (23%) | 25 (43%) | 0.08 |
| Dipeptidyl Peptidase IV Inhibitors | 2 (2%) | 0 | 2 (6%) | 0.18 | 0 | 2 (3%) | >0.99 |
| Sodium-glucose cotransporter 2 inhibitors | 2 (2%) | 1 (2%) | 1 (3%) | >0.99 | 0 | 2 (3%) | >0.99 |
| Glucagon-like peptide-1 receptor agonists | 0 | 0 | 0 |  | 0 | 0 |  |
| Insulin | 37 (44%) | 18 (38%) | 19 (53%) | 0.16 | 12 (46%) | 25 (43%) | 0.79 |
| Insulin + metformin | 9 (11%) | 7 (15%) | 2 (6%) | 0.29 | 0 | 9 (16%) | 0.05 |
| Insulin + insulinotropic agents | 7 (8%) | 3 (6%) | 4 (11%) | 0.46 | 2 (8%) | 5 (9%) | >0.99 |
| Metformin + alpha-glucosidase inhibitors | 11 (13%) | 9 (19%) | 2 (6%) | 0.11 | 0 | 11 (19%) | 0.02 |

Primary composite end-point event contains admission to ICU, or mechanical ventilation, or death. Data are median (IQR) and n (%). *P* values are from χ² test, Fisher’s exact test, or Mann-Whitney U test. 2019-nCoV=2019 novel coronavirus.
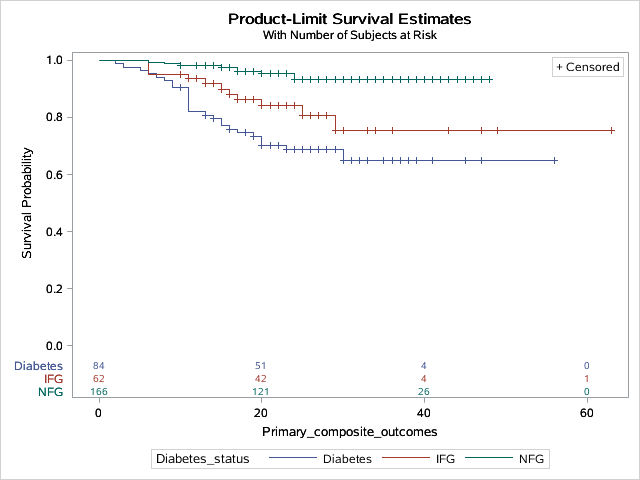

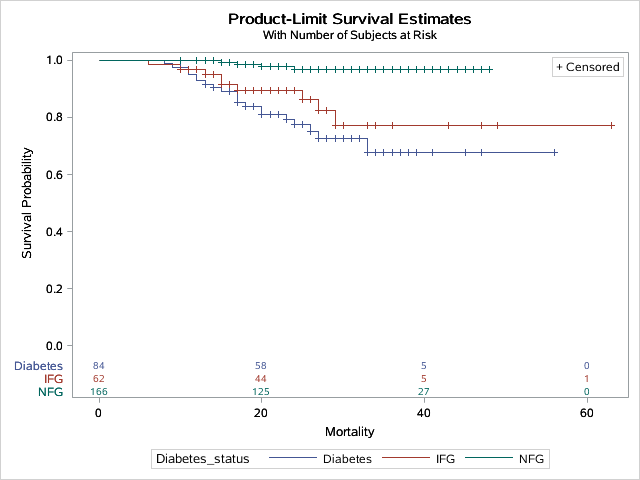

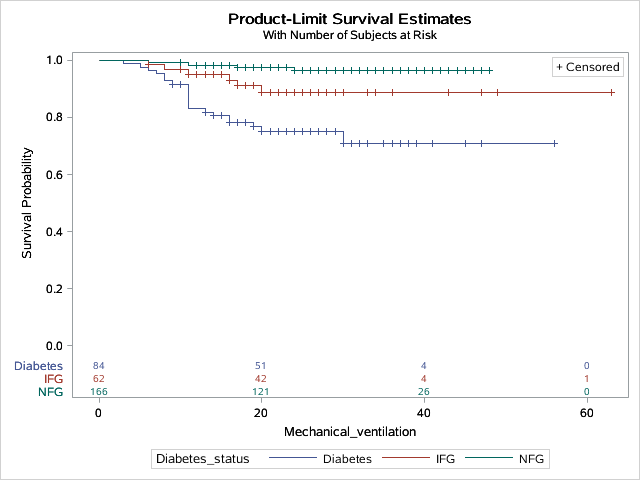


1. Primary composite endpoint B. Mortality C. Mechanical ventilation

**Supplementary Figure 1 Survival curves of patients with COVID-19 according to diabetes status for the primary composite endpoint (A), mortality (B) and mechanical ventilation (C).**

**References**

1. Wu Z, McGoogan JM. Characteristics of and Important Lessons From the Coronavirus Disease 2019 (COVID-19) Outbreak in China: Summary of a Report of 72314 Cases From the Chinese Center for Disease Control and Prevention. *JAMA.* (2020). doi: 10.1001/jama.2020.2648.

2. Metlay JP, Waterer GW, Long AC, Anzueto A, Brozek J, Crothers K, et al. Diagnosis and Treatment of Adults with Community-acquired Pneumonia An Official Clinical Practice Guideline of the American Thoracic Society and Infectious Diseases Society of America. *American Journal of Respiratory and Critical Care Medicine.* (2019) 200:E45-E67. doi: 10.1164/rccm.201908-1581ST.

3. Association AD. Diagnosis and classification of diabetes mellitus. *Diabetes Care.* (2014) 37:S81–S90.

4. WHO. Clinical management of severe acute respiratory infection when Novel coronavirus (nCoV) infection is suspected: interim guidance.(2020). Available online at: https://www.who.int/publications-detail/clinical-management-of-severe-acute-respiratory-infection-when-novel-coronavirus-(ncov)-infection-is-suspected. (accessed May 27, 2020).

5. Kidney disease: improving global outcomes (KDIGO) acute kidney injury work group. KDIGO clinical practice guideline for acute kidney injury. (2012). Available online at: https://kdigo.org/wp-content/uploads/2016/10/KDIGO-2012-AKI-Guideline-English.pdf (accessed May 27, 2020).

6. Huang C, Wang Y, Li X, Ren L, Zhao J, Hu Y, et al. Clinical features of patients infected with 2019 novel coronavirus in Wuhan, China. *Lancet.* (2020) 395:497-506. doi: 10.1016/S0140-6736(20)30183-5.

7. Gao C, Wang Y, Gu X, Shen X, Zhou D, Zhou S, et al. Association Between Cardiac Injury and Mortality in Hospitalized Patients Infected With Avian Influenza A (H7N9) Virus. *Crit Care Med.* (2020) 48:451-8. doi: 10.1097/CCM.0000000000004207.

8. Graham DJ, Staffa JA, Shatin D, Andrade SE, Schech SD, La Grenade L, et al. Incidence of hospitalized rhabdomyolysis in patients treated with lipid-lowering drugs. *Jama-Journal of the American Medical Association.* (2004) 292:2585-90. doi: 10.1001/jama.292.21.2585.

9. Fayfman M, Pasquel FJ, Umpierrez GE. Management of Hyperglycemic Crises: Diabetic Ketoacidosis and Hyperglycemic Hyperosmolar State. *Med Clin North Am.* (2017) 101:587-606. doi: 10.1016/j.mcna.2016.12.011.

10. Ben-Ami H, Nagachandran P, Mendelson A, Edoute Y. Drug-induced hypoglycemic coma in 102 diabetic patients. *Archives of Internal Medicine.* (1999) 159:281-4. doi: 10.1001/archinte.159.3.281.
